# The impact of a structured handover checklist for intraoperative staff shift changes on effective communication, OR team satisfaction, and patient safety: a pilot study

**DOI:** 10.1186/s13037-021-00299-1

**Published:** 2021-07-18

**Authors:** Ebrahim Nasiri, Mojgan Lotfi, Seyyed Muhammad Mahdi Mahdavinoor, Mohammad Hossein Rafiei

**Affiliations:** 1 Assistant Professor of Traditional Medicine, Department of Anesthesiology and Operating Room, Faculty of Allied Medical Sciences, Mazandaran University of Medical Sciences, Sari, Iran; 2 Associate Professor of Nursing Education, Department of Medical Surgical Nursing, Faculty of Nursing and Midwifery, Sina Hospital, Tabriz University of Medical Sciences, Tabriz, Iran; 3 Undergraduate Bachelor Student of Surgical Technology, Department of Anesthesiology, Operating Room and Emergencies, School of Allied Medical Sciences Mazandaran University of Medical Sciences, Sari, Iran; 4 MSc Student in Surgical Technology, School of Allied Medical Sciences, Student Research Committee, Mazandaran University of Medical Sciences, Sari, Iran

**Keywords:** Patient Safety, Operating Room, Shift Change, Quality of Care

## Abstract

**Objective:**

Handover without a structured format is prone to the omission of information and could be a potential risk to patient safety. We sought to determine the effect of a structured checklist on the quality of intraoperative change of shift handover between scrubs and circulars.

**Methods:**

We conducted a control intervention study on operating room wards of two teaching hospitals from 20 Feb to 21 Nov 2020. This research was conducted in three stages as follows: assessing the current situation (as a group before the intervention), performing the intervention and evaluating the effect of using a checklist on handover quality after the intervention in two groups: with and without checklist. We examined the quality of handover between scrub and circular personnel in terms of handover duration and quality, omission of information and improvement in OR staff satisfaction.

**Results:**

A total of 120 handovers were observed and evaluated. After intervention in the group using the checklist, the percentage of information omission in surgical report was decreased from 19.5 to 12.1% between scrubs (P < 0.00) and from 16.8 to 14.1% between circulars (P < 0.03). Also, in the role of scrub, the mean overall score of handover process quality was significantly higher after the intervention (x̄ = 7 ± 1.5) than before it (x̄ = 6.5 ± 0.9) (p < 0.02). In the role of circulating, despite the positive effect of overall score checklist, no significant difference was observed (p < 0.08). The use of checklist significantly increased the handover duration between scrubs (p < 0.03) and circulars (p < 0.00). The overall mean percentage of handover satisfaction increased from 67.5% before the intervention to 85.5% after the intervention (p < 0.00).

**Conclusion:**

The implementation of a new structured handover checklist had a positive impact on improving the quality of communication between the surgical team, reducing the information omission rate and increasing the satisfaction.

## Introduction

The transfer of essential information and patient care responsibilities from one health care provider to another is a crucial component of communication in health care, which is known as handover. Effective communication during handover is among the main priorities of health care. In handover, accurate transfer of information regarding the status and care plan safeguards the safety and continuity of patient care [1, 2].

On average, 4.8 handover transfers are performed per patient in an operating room [3]. Moreover, the surgical team is responsible for patient's safety during surgical care because the patient cannot inform the team in case there is incorrect information, which indicates the importance of attempts to prevent mistakes in the operating room [4].

Studies have shown that 21–65% of errors and mistakes of patient care during surgery are related to communication problems during handover [5]. Communication errors often occur every seven to eight minutes in the operating room. In 90% of cases, these errors have adverse effects on surgery in multiple ways, including delays, surgical errors, waste of resources, team tension, omission of information, or unfavorable events [6–8].

The time of shift delivery during surgery is among critical mattes in which irreversible errors can occur; given that the operating room staff work according to the shift schedule, it is possible to change the shifts of scrubs and circulars when the surgery is prolonged. At the time of delivery, information are transmitted concerning patient's characteristics, medical records and illnesses, as well as equipment considerations (including plate location, time and pressure of the tourniquet, etc.). Besides, information on countable items, location of seizures, samples and drugs on the sterile field and so on should be conveyed. Defective transmission of such information can endanger the safety and even the life of patients [9, 10].

In various studies, several factors such as lack of personnel training, absence of standardization, non-existence of face-to-face communication, passive transfer (without the possibility of interactive discussion), interruptions and time constraints have been mentioned as barriers to successful handover [11–13].

For this reason, international organizations and agencies all over the world have stressed the need for standardization of this part of care, so that Joint Commission stated the standardization of communications as a priority of health care organizations in 2012. The World Health Organization (WHO) has also recommended and supported the development of a structured process. Health Research and Quality Agency (AHRQ) also calls for formalization of communication processes, which can help decrease errors at this critical time of patient care process [14–17].

In our literature review, no study was found to specifically address the impact of standardization of intraoperative change-of-shift handovers. The descriptive cross-sectional study of Hawthorne et al. examined the application of SBAR standard model (status, background, assessment, recommendation) in communication between surgical team six years after training [18]. The study of Johnson et al. also introduced only the standard SWITCH (S: Surgical procedure, W: Wet (i.e., fluids), I: Instruments, T: Tissue (i.e., specimen), C: Counts and H: Have you any questions?) checklist for surgical shift delivery but did not intervene to determine its effectiveness [10].

Therefore, this study was conducted to evaluate the use of a structured checklist during shift delivery at the time of surgery. We hypothesized that the implementation of this intervention would reduce the percentage of information omission and increase the communication quality of handover process. We also assumed that improving communication between the surgical team could increase their satisfaction.

## Methods

### Study design

This study is was performed as an intervention study from 20 Feb to 21 Nov 2020 in the operating room wards of Sina and Imam Reza hospitals affiliated with Tabriz University of Medical Sciences, Iran. The levels of evidence for this study was Level 3: “Evidence obtained from well-designed controlled trials without randomization”. We sought to determine the effect of a structured checklist on quality of intraoperative change of shift handover between scrubs and circulars.

At the beginning of the current project, there was no standard program for shift change handover between circulars and scrubs during surgery. To achieve the objectives of the research, first we reviewed the literature on the Internet by searching for the following keywords: Operation room, handover, perioperative, handover, shift report, sign out, shift change, information, checklist; and the guidelines and standards related to documentation in the operating room were also investigated. According to reviews, the SWITCH checklist was found for the change of shift handover during surgery. The SWITCH checklist consists of six sections (S: Surgical procedure, W: Wet (i.e., fluids), I: Instruments, T: Tissue (i.e., specimen), C: Counts and H: Have you any questions?). Each of the SWITCH acronyms enables additional sub-categories such as drugs in the Wet group that allows surgical team members to make specific connections to different tasks and meet their handover needs appropriately. To complete the checklist and adapt it to the present situation, the SWITCH checklist was revised by reviewing the guidelines provided by AORN (Association of periOperative Registered Nurses) and AST (Association of Surgical Technologists) associations; subsequently, based on the opinions of faculty members and specialists in the field, final editing was performed and examined for validity and reliability.

This study was performed in three stages as follows. The first stage was to evaluate the current situation in terms of handover quality before the intervention, the second stage was to perform the intervention by training, introducing a checklist and its application in shift delivery during surgery, and the third stage was to assess the effect of using a checklist on handover quality after the intervention.

Inclusion criteria in this study were all circulating and scrub personnel with at least one year of experience in the relevant field, and exclusion criteria were people who had previously been trained on how to deliver shifts by a checklist or another shift delivery program or who did not wish to continue the study.

Before the intervention, we observed and evaluated sixty cases of handover for the initial assessment, which were defined as Group A (no knowledge or use of checklist). After the intervention, sixty handovers were reviewed as group B (as a control group with knowledge but no use of checklist) and group C (with the use of the checklist attached on operation room wall). For all handovers in Groups A and B, the personnel were instructed to give a handover as usual. For all handovers in Group C, the staff were told to handover the patient using the checklist.

### Intervention

In order to conduct the study, after receiving the license, ethical code and performing the administrative steps, we recruited 40 operating room staff working in the two hospitals under study by explaining the objectives of research to them.

We held a training session in the operating room of both hospitals for 30 min on two separate days. Those who were not present in the work shift on that day were trained individually, and all questions of staff were answered and ambiguities resolved. Then, we instituted a one-week interval for normalizing the researcher's presence in the ward as a member of the group and eliminating its effect on staff behavior. During this period, the researcher was present in the operating room as a member of the group but collected no data. After one week, the researcher was present in the ward every day before shift delivery and evaluated the handover with the relevant assessment forms. Also, all personnel were unaware of the specific criteria of data collection.

Samples were taken by convenience sampling, the sample size was calculated based on similar previous studies[1]. 120 handover was calculated. In order to properly distribute the samples and cover the types of surgeries in both circular and scrub roles, sampling was performed proportionally according to the type of surgery and the role of personnel (circular and scrub). Meanwhile, sampling was done when all participating members of the handover expressed their satisfaction of the study.

### Data collection tools

The standard CEX-instrument care delivery assessment tool was used to evaluate the quality of handover process [19, 20]. This tool is rated on a scale of 1–9, which is used to evaluate the information transfer process in six areas, including environment, organization, communication skills, content, clinical judgment, professionalism and overall delivery competence. The Persian version of "CEX-instrument" was prepared based on the standard translation and equivalence process steps.

To assess the quality of handover content and the amount of omitted information transfer, the data collection form extracted from 17 items provided by the Association of Surgical Technologists (AST) was used to deliver the standard shift [9].

Since staff satisfaction is one way to evaluate the results of using a standard method, we used the edited questionnaire of Petrovic study after confirming its validity and reliability [21]. In this questionnaire, the level of satisfaction is measured in the form of 10 questions using a five-point Likert scale, of which a score of 1 means complete disagreement and a score of 5 denotes full agreement.

### Analysis

We used SPSS (version 26) statistical software for data analysis. Descriptive indicators were used to determine the mean, standard deviation, and Kolmogorov–Smirnov method was employed to examine the normal distribution of data. In case of normal and abnormal data distribution, t-test and Mann–Whitney test was used, respectively. P < 0.05 was considered as significance level in this study.

## Results

A total of 120 handovers were observed during intraoperative change-of-shift between circular and scrub members of the surgical team. The number of participants in these handovers was 40. The mean and standard deviation of participants’ ages were 32.2 ± 5.6 years. Other demographic information of personnel is given in Table 1.

**Table 1:** Demographic information in the two hospitals surveyed

Characteristic		Hospital-1(n = 22)	Hospital-2(n = 18)	P-value
Age (years)		32.8(SD = 7.31)	30.02(SD = 4.96)	T test: 0.36
Sex	Male	8	6	Chi square:
	female	13	12	0.32
Work history (years)		10(SD = 6.85)	9.2(SD = 7.22)	T test: 0.77
Education level	Associate degree	4	5	Chi square: 0.89
	Bachelor’s degree	17	13	
Type of employment	Training course	6	5	Chi square: 0.76
	Contractual	0	3	
	Permanent	15	10	

Hospital-1: Sina, Hospital-2: Emam Reza

### Duration of handover

Before the intervention, the mean handover delivery time between circulars and between scrubs was x̄ = 67.7 ± 19 and x̄ = 92.7 ± 39 s, respectively. The use of checklist significantly increased the handover time between scrubs (two-sample t-test: p < 0.03) and circulars (two-sample t-test: p < 0.00). There was no significant difference between B and C groups (two-sample t-test: p < 0.8). Information about surgeries and the average duration of shift handover delivery are presented in Table 2.

**Table 2:** Duration of handover in observed surgeries

Type of surgery	Before intervention **Group A** (X̄ ± SD)	After intervention	
		**Group B**(without checklist)	**Group C**(with checklist)
Gynecology			
C to C	64 ± 21	89 ± 11*	84 ± 8*
S to S	110 ± 24	136 ± 34*	153 ± 27*
Neurosurgery			
C to C	42 ± 12	59 ± 16	74 ± 9*
S to S	114 ± 21	142 ± 21*	151 ± 28*
General			
C to C	105 ± 24	31 ± 23*	147 ± 18*
S to S	142 ± 35	144 ± 28	160 ± 43*
Orthopedic			
C to C	35 ± 12	34 ± 16	28 ± 16
S to S	45 ± 13	51 ± 17	49 ± 9
Urology			
C to C	62 ± 9	-	-
S to S	54 ± 14		
All surgery			
C to C	67 ± 19	76 ± 34*	85 ± 39*
S to S	92 ± 39	118 ± 21*	128 ± 21*

*C:* Circular, *S:* Scrub

^*^P < 0.05; significant difference from before intervention

### Handover process quality

**Fig. 1:**
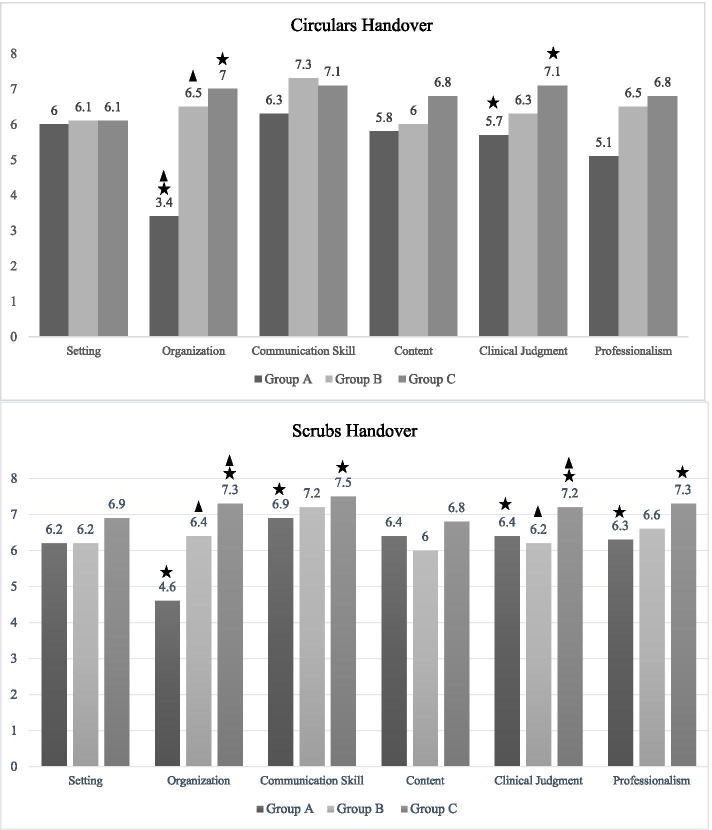
Comparison of handovers process quality scores In Group **A** (Before intervention), Group **B** (Without checklist) and Group **C**

The review results of handover process quality include six areas: environment, organization, communication skills, content, clinical judgment, and professionalism. The findings showed that the overall score of handover process quality was x̄ = 7 ± 1.5 in group C (with checklist) after the intervention in the role of scrub, which was significantly higher than that before the intervention (x̄ = 6.5 ± 0.9) (p < 0.02). In the role of circulating, despite the positive effect of checklist in all six areas, there was no significant difference in the overall score between before (x̄ = 5.9 ± 1.6) and after the intervention (x̄ = 6.9 ± 0.7) (p < 0.08). Comparison of the scores of handover process quality before and after the intervention is presented in Fig. 1. As can be seen from the diagram, the most significant increase in handover quality after the intervention was in the areas of communication skills, organization and professionalism.

### Handover content quality

**Fig. 2:**
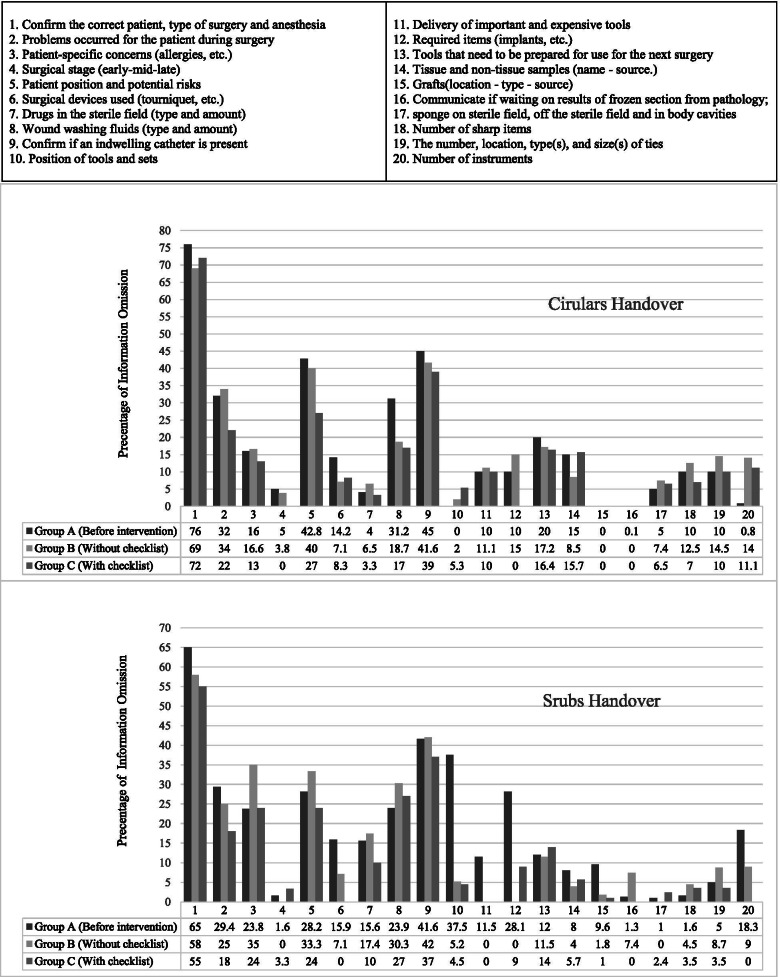
Information omissions pre and post intervention

On average, the percentage of information omission related to shift delivery during surgery before the intervention between two circulars subjects was x̄ = 17.3 ± 19.4 (Min = 0, Max = 76), which was x̄ = 18 ± 16.3 (Min = 0, Max = 65) between two scrub subjects. Details of the percentage of information omission in the handover before and after the intervention is given in Fig. 2.

After the intervention, the percentage of information omission in group B (handover without checklist) between two circulars was x̄ = 16.9 ± 17 (Min = 0, Max = 69) and between two scrubs was x̄ = 15 ± 16.6 (Min = 0, Max = 58). Statistical analysis showed no significant difference between circulars (p < 0.79, t = 0.3, df = 19) and scrub handover (p < 0.1, t = 1.6, df = 19) in group B with before the intervention.

After intervention in group C (handover with checklist), the percentage of information omission between two circulars was x̄ = 14 ± 16.8 (Min = 0, Max = 72) and between two scrubs was x̄ = 12.1 ± 14.7 (min = 0, Max = 55). Statistical analysis showed a significant difference between circulars (p < 0.03, t = 2.2, df = 19) and scrubs handover (p < 0.00, t = 3.3, df = 19) in group C with before the intervention. After the intervention, there was significant difference between B and C groups, the percentage of information omission in handover reports during surgery between the circulars (p < 0.03, t = 2.3, df = 19) and between scrubs handover (p < 0.02, t = 2.5, df = 19).

### Staff satisfaction from handover

A total of 30 handover satisfaction survey questionnaires were completed out of 40 personnel participating in this study whose handover was surveyed. The overall mean percentage of handover satisfaction increased from 67.5% before the intervention to 85.5% after the intervention, which was also statistically significant (p < 0.00, t = 3.5, df = 18). Table 3 shows the percentage of answers to questions before and after the intervention.

**Table 3:** Surgical Team Satisfaction from handover

	pre		post	
Items	**n**	**agree and strongly agree%**	**n**	**agree and strongly agree%**
1. I was satisfied with the handover for this patient	20	65%	28	93%
2. I could hear all of the report	24	85%	28	93%
3. I received information about potential problems that could arise in this patient	26	85%	26	86%
4. I received information on things that I need to follow up	22	75%	20	66%
5. The patient’s condition matches what I get in report	20	65%	28	93%
6. I was clear as to when the handover actually started and ended	27	90%	28	93%
7. Shift delivery reports allow me to prioritize my tasks	17	55%	24	80%
8. Immediately after nurse-to-nurse shift report, I am able to communicate with physicians regarding patient care	20	65%	19	63%
9. The length of report is an effective use of my time	12	40%	28	93%
10. Mistakes in patient care and equipment rarely occur in the current shift delivery process	15	50%	28	93%
Total mean		67.5%		85.5%

## Discussion

We found that having a checklist in the operating room improves the quality of intraoperative change of shift handover for scrubs and circulars, although the increasing quality of handover process was only significant for scrubs. Also, the presence of a checklist in the operating room significantly reduced the omission percentage of information in the shift report during surgery in both circulars and scrubs compared to before the intervention.

After performing the intervention and introducing the contents of checklist and the items to be transferred during the shift delivery at the time of surgery, an increase in handover duration was seen in both B and C groups (with and without checklists) along with the improvement of data transfer status. The difference between this increases of time in the checklist group before the intervention was 35.3 s among scrubs and 17.3 s among circulars. Researchers in similar studies have reported different perceptions of increasing or decreasing handover duration after intervention and introduction of a checklist [1, 22–24]. For example, in the study of Salzwedel et al., the increase in handover time, which was less than 1 min in their study, was considered to be short-lived, and this slight increase in time was compared to the improvement in the status of information transfer as well as quality of positive patient care [1]. In contrast, in the study of Catchpole et al., a decrease was observed in the handover duration by intervening and introducing a checklist, citing the structuring of handover and defining responsibilities [23].

Increasing handover duration in this study may not be economically viable at first glance; however, this slight increase over time can be ignored because of the positive aspects of using a checklist to reduce the amount of omitted information that improves the quality of handover process and ultimately increases the quality of patient care.

The quality of handover process in this study was evaluated in two aspects: environment (noise and interruptions created) and behavior (organization, communication skills, clinical judgment and professionalism). According to the results of environmental aspect, no change in the improvement of situation was observed after the intervention. In other similar studies, for example, in the study of Lo concerning the handover delivery of medical attending, or in the study of Joy investigating the patient handover between cardiac surgery department and ICU, improvement in the environment and interruptions during the handover has been reported after the application of checklist [25, 26].

However, the operating room environment is full of sound generating sources (sound of electrical equipment, air conditioning system, moving devices and tools, etc.) [27, 28]. These sounds can have adverse effects on correct transmission of information. The circulating and scrub roles during surgery are such that multiple interruptions during handover are inevitable. Therefore, in this study, after the intervention, we did not observe any significant change in the environment of handover process. However, behavioral intervention in the two areas of handover organization and communication skills showed better results, which was made possible due to the existence of a checklist. In other words, the existence of a checklist enabled the surgical team to resume the process of transferring information from the part that had been stopped despite numerous interruptions. On the other hand, the checklist improved communication skills by approaching the deliverer and recipient to each other. This occurred during the handover, and eventually reduced the lost information of handover reports despite lack of change in the scope of environment after the intervention.

Our findings support the study hypothesis that we can increase the quality of handover content during surgery by introducing a relatively structured and regular method.

Hanley cites three potential reasons for handover errors: interruptions during hand-off, lack of standard format, and unreliability of the sender and receiver of the information to be transmitted [29]. In this study, by performing the intervention despite the inability to control interruptions during handover via introducing the standard format and teaching the information to be transferred during shift delivery, we observed a reduction in information omission percentage as well as improved quality of handover content in both circular and scrub roles.

The use of a standard model for handover in similar studies also improved the information transfer. In the study of Ding et al., the patient handover between neurological intensive care unit and neurological department after the intervention showed a reduction in handover errors from 18.8% to 5.7% [30].

In the study of Craig et al., the percentage of information omission was reduced from 36.8% before the intervention to 15.7% after it through introducing the standard format for handover among cardiology and ICU staff [28]. In Mitchelle's study on the same statistical population, the amount of information lost after the intervention was reduced from 26 to 18% [29]. In Negpal’s study, the amount of information lost after using the standard checklist was reduced from 9 to 3% [2].

Participants' satisfaction is another important element in evaluating the success of an intervention. In the present study, handover satisfaction increased from 67.5% to 85.5% using the checklist, which was consistent with previous studies that successfully implemented a standard handover process. In the study of Petrovic et al., the satisfaction of recovery nurses with handover increased from 73.8% to 92% after using the checklist for patient transfer [21]. In a study by Johnson et al., nurses' satisfaction with patient's handover after the introduction of checklist increased from a total score of 21.7 to 24 [31]. In the study of Kazemi et al., which was conducted with the aim of evaluating the effect of delivery of nurses' shifts in patient's bedside with patient's participation, total satisfaction score increased from 81.6 to 93 [32].

### Limitations

One of the limitations of this study was its small sample size. With more samples, better conclusions and a more accurate p-value for weaving is possible. Therefore, it is recommended that future studies with a higher sample size examine the impact of a checklist on the quality of handover during shift delivery. Other limitations of this study was the evaluation of shift-change handover during surgery with the presence of a researcher in the operating room, which could cause Hawthorne effect and influence the results. However, this effect was limited because the assessor did not communicate with the surgical team during handover. Also, the assessor was present in both periods before and after the intervention. On the other hand, the researcher was not a member of the operating room staff and had no control over them. Nevertheless, the presence of an assessor was necessary to evaluate the handover in the operating room, and a camera can be used in subsequent studies to assess handover and reduce the effect. Another limitation was the COVID-19 pandemic during the study, which could affect the research process and was beyond the researcher's control, although this effect was the same in both B and C groups.

## Conclusion

This study showed that the use of a standard format among the surgical team in health centers had a positive effect on increasing quality of communication process and reducing the omission percentage of information as well as increasing the satisfaction of surgical team during shift delivery at the time of surgery. Therefore, we recommend standardized tools in critical situations such as shift change handover during surgery after receiving the necessary training. We also suggest that in future studies, the effect of intervention and the use of a standard checklist on the outcome of possible injuries to the patient should be investigated.

## Data Availability

The datasets used and/or analyzed during the current study are available from the corresponding author on reasonable request.
